# Microengineering Design for Advanced W-Based Bulk Materials with Improved Properties

**DOI:** 10.3390/nano13061012

**Published:** 2023-03-11

**Authors:** Magdalena Galatanu, Monica Enculescu, Andrei Galatanu, Dorina Ticos, Marius Dumitru, Catalin Ticos

**Affiliations:** 1 National Institute of Materials Physics, Atomistilor Street 405 A, Magurele, 077125 Ilfov, Romania; 2 National Institute for Laser, Plasma and Radiation Physics, Atomistilor Street 409, Magurele, 077125 Ilfov, Romania

**Keywords:** materials for fusion applications, high-heat-flux materials, thermophysical properties, 6 MeV electron irradiation

## Abstract

In fusion reactors, such as ITER or DEMO, the plasma used to generate nuclear reactions will reach temperatures that are an order of magnitude higher than in the Sun’s core. Although the plasma is not supposed to be in contact with the reactor walls, a large amount of heat generated by electromagnetic radiation, electrons and ions being expelled from the plasma will reach the plasma-facing surface of the reactor. Especially for the divertor part, high heat fluxes of up to 20 MW/m^2^ are expected even in normal operating conditions. An improvement in the plasma-facing material (which is, in the case of ITER, pure Tungsten, W) is desired at least in terms of both a higher recrystallization temperature and a lower brittle-to-ductile transition temperature. In the present work, we discuss three microengineering routes based on inclusions of nanometric dispersions, which are proposed to improve the W properties, and present the microstructural and thermophysical properties of the resulting W-based composites with such dispersions. The materials’ behavior after 6 MeV electron irradiation tests is also presented, and their further development is discussed.

## 1. Introduction

Obtaining energy from fusion reactions might be the only long-term sustainable solution for the increasing world energy demand [1]. In a fusion reactor, such as ITER or DEMO, the plasma used to generate nuclear reactions will reach temperatures that are an order of magnitude higher than in the Sun’s core, which is close to 200 million degrees [1]. Although this plasma is not supposed to be in contact with the reactor walls, a large amount of heat generated by electromagnetic radiation, electrons and ions being expelled from the plasma will reach the plasma-facing surface of the reactor. Thus, even in normal operating conditions, a steady heat flux of about 10 MW/m^2^ [2] is expected for the divertor (about 5% of the total exposed surface), which will extract, in fact, about 15% of the energy produced in the reactor. A tokamak divertor is, as its name already suggests, the part where the magnetic flux lines are open, allowing for unsuitable particles from plasma to escape [3,4]. Therefore, the heat load at the divertor level will be even higher at the beginning and the end of each plasma cycle, around 20 MW/m^2^, albeit for a short time. Plasma disruptions and turbulences, if not mitigated by magnetic field control, can even reach half a GW/m^2^ [5]. Thus, at least the plasma-facing part of the divertor should be a refractory material, able to withstand temperatures in normal operating conditions of about 1000 °C and, in particular situations, even over 2500 °C [5]. Then, the large amount of heat should be efficiently transferred to the cooling system, implying that the divertor materials require high thermal conductivity. As it is a general requirement for fusion reactor materials, the divertor should also be made only from low-activation elements, criteria that, when considered together, restrict the number of acceptable materials to only a few elements. Tungsten (W) was selected as the main divertor armor material for ITER and is the “base-line” option for DEMO due to its high melting temperature, high physical sputtering yield, high thermal conductivity and low Tritium retention. These characteristics are advantages over low-Z elements (e.g., C), which have lower sputtering yields and higher Tritium retention rates. On the other hand, W will be affected by neutron irradiation and also by subsequent transmutation and gas (H, He) formation. These considerations exclude W in the first step as a material for structural applications, but it offers a good compromise solution for an armor material, at least in the case of the ITER reactor [6], where a lower neutron irradiation dose is expected. As a consequence, the ITER reactor will be built with a full W divertor armor [7,8]. In contrast, in DEMO (which will be the first demonstrative reactor able to provide energy to the grid), higher temperatures and irradiation doses are foreseen, which demand a more complicated design [9] and, if possible, better materials [10]. In this context, other W shortcomings become more relevant:

(i) W is a brittle material: i.e., it has a high brittle-to-ductile transition temperature (BDTT) of about 300–400 °C [11]. As a result, W is prone to fail at low temperatures as a result of brittle fracture, which is a concern even for armor materials [12].
(ii) W has a low (4.5 × 10^−6^ K^−1^) thermal expansion coefficient (CTE), which generates problems related to its joining to structural materials, which in turn have much higher CTEs (10 ÷ 17 × 10^−6^ K^−1^ for steels and 16 ÷ 18 × 10^−6^ K^−1^ for Cu alloys). The CTE mismatch between armor and structural materials is a serious concern since thermal fatigue in the divertor joints might lead to severe failures.
(iii) W oxidizes easily even at modest temperatures (600 °C), and even a small cooling-fluid accident might compromise the entire reactor for a long time. To address this problem, the so-called self-passivation or “smart W alloys” might provide a solution [13]. Such materials contain elements (Cr, Y) that create an oxide barrier limiting O penetration in the W volume. However, the improved oxidation resistance comes at the expense of a much lower thermal conductivity (100 W/m/K or even lower), which is not suitable for the divertor armor.

Taking these considerations into account, the optimal operating temperature for W might be defined with a lower limit derived from both the BDTT and recovery temperature under neutron irradiation at around 800 °C and an upper limit derived from recrystallization considerations at about 1200 °C [14]. On the other hand, for a low-temperature cooling concept (as in ITER, with the cooling fluid being water), the heat sink materials are Cu-based alloys [15], such as CuCrZr or GlidCop. Such alloys can operate at a maximum temperature of 300–350 °C. Using new materials such as oxide-dispersion-strengthened (ODS) Cu alloys [16,17] or W-reinforced Cu [18], the heatsink operating temperature might be raised up to about 400–450 °C. Again, recovery under neutron irradiation imposes a low-temperature limit for Cu-based materials at about 200 °C. Obviously, the operating temperature windows of the armor and heat sink materials do not overlap. To improve the divertor component behavior in terms of both the operating temperature window and CTE mismatch, several interface materials have been proposed, ranging from pure soft Cu to functional W-Cu graded composites [19,20] and thermal break (or thermal barrier) materials such as Cu-based composites [21,22,23]. Such interfaces respond well to the CTE mismatch problem but fail to completely solve the operating temperature window mismatch. They also make the production of the divertor components more difficult and expensive. An alternative solution is to find new structural materials, such as W-laminates (layered composites made from alternating thin foils of W and other metals) [24,25] or HEA high-entropy alloys [26,27]. Again, these solutions are either far from technological maturity or might further increase production costs to higher levels. However, another alternative approach is to improve W armor materials to better cope with the available structural materials. A focused approach on a particular weak point of a material might be successful, as has been demonstrated by the accelerated development of self-passivating W “smart alloys” [27,28], which are able to solve an important issue for the first wall armor, although remaining non-applicable to the divertor, where high thermal conductivity values are essential.

In order to design a new approach to improving W, it is important to start with the processing of W, which is already used on an industrial scale. Its production includes the high-temperature sintering of blocks, which are then hot-rolled (i.e., at temperatures above the recrystallization temperature of W) and transformed into large plates with thicknesses down to a few centimeters. The rolling temperature decreases with decreasing thickness, and cold-rolling processes are also sometimes used, e.g., to produce thin foils (down to 0.05 mm).

In contrast to other bcc materials, the W microstructure (grain size, texture) plays a major role in its mechanical behavior, particularly its intergranular fracture behavior, which is responsible for the high BDTT of W. Taking into account the high-temperature processing of W, it was initially assumed that the inherent impurities that accumulate between W grains induce intergranular fracture [29]. More recent results have shown that the impurity effect is much smaller than expected [30,31], while low cohesion at high-angle (high means more the 15°) grain boundaries might be the decisive factor in W fracture [32,33,34,35,36]. This assumption is also supported by the fact that severely deformed W (e.g., cold-rolled) has a highly anisotropic behavior, with two transitions: one from brittle to delamination and one from delamination to ductile [11]. Thus, one can assume that using W powders with suitable grain dimensions and different types of ceramic or metallic dispersions in an SPS (Spark Plasma Sintering)-based processing route might be able to both increase the recrystallization temperature and decrease the DBTT transition of W while maintaining a high value of thermal conductivity. More specifically:

- Severe plastic deformation of W produces ultrafine grains or texture [37,38], which decreases the W DBTT, even to values well below room temperature.
- Grain growth and recrystallization can be shifted to higher temperatures by nanometric dispersions, but to the best of our knowledge, no systematic study has been performed to evaluate the effect of the dispersion type and dimensions. Additionally, in some cases, the effects are complex, i.e., a decrease in the brittle-to-delamination transition temperature but an increase in the delamination-to-ductile transition temperature [11,39].

In this work, aiming to improve W in terms of both a higher recrystallization temperature and a lower brittle-to-ductile transition temperature, three microengineering routes based on inclusions of nanometric dispersions are investigated, and the further development of the materials is discussed.

## 2. Materials and Methods

### 2.1. Design Approach

The experimental work is based on the fast-forward screening of some ceramic and metallic candidate dispersions. Considering the high-temperature processing of W-based materials and the high oxidation rate of W (producing W oxides, which are unstable when the temperature is changing), we excluded oxide-type ceramics from this study. Thus, the focus was on 3 types of nanometric dispersions:

- Carbides such as SiC or ZrC and diamond, with the last one most likely producing W-C. In fact, Z.M. Xie et al. have shown that hot-rolled W with ZrC exhibits improved malleability [40], while SPS-ed W with nanometric dispersions of ZrC [41] shows better recrystallization behavior and improved mechanical properties.
- Metals that can interact with W during SPS processing, such as Cr. Using nanometric Cr powders, we expect a limited reaction at the bigger W grain borders, as depicted in the diagram below:
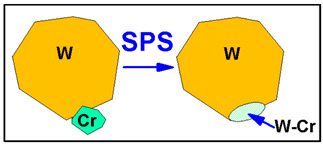

- Metals that melt during SPS but do not react strongly with W, such as Fe. Small amounts of such metals can be included in W without causing the entire sample to melt in SPS processing or in further heating above the Fe melting temperature. Here, we might also expect a “pseudo-ductility effect” created by the coating of W grains with Fe, as observed in the W fibers/W matrix in the case of coated fibers [42].

### 2.2. Materials Processing

**Figure 1:**
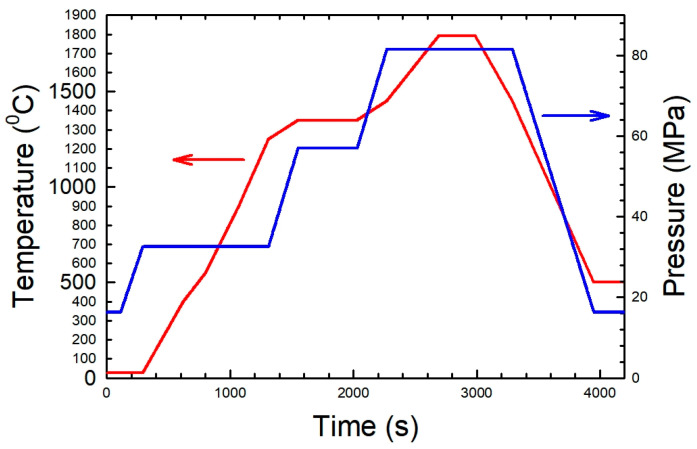
SPS cycle used to produce the samples for fast-forward screening.

The samples were consolidated by SPS using a mixture of 75% 40 μm and 25% 800 nm W powders. Such compositions are expected, based on previous results [43], to provide higher densities. The W powders, together with nanometric ceramic or metallic dispersions, were mixed under a controlled atmosphere in a glovebox (Ar, with <0.5 ppm O_2_ and <0.8 ppm H_2_O) by hand, then closed in Agate-coated vials with a small number of ϕ 5 mm Agate balls and mechanically mixed with a planetary ball mill at 50 rpm for 5 min. Then, the powders were transferred to molds, also inside the glovebox. An SPS cycle with 3 steps was used (see Figure 1), which, for pure W powders, produces samples with densities above 95%. Disc samples with a 12.5 mm diameter and ~2 mm thickness were produced using ceramic and metallic dispersions: SiC (20 nm), ZrC (20 nm), diamond (10 nm), Cr (40 nm) and Fe (60 nm). A summary of the materials produced can be seen in Table 1.

In order to speed up the investigations, a fast-forward screening was performed by producing several samples with different amounts of dispersions from the first-candidate composites. The main selection criteria are the sample density (at least 95% of the theoretical density in order to make the other investigations relevant), the microstructure (to determine whether the dispersions are located as expected) and the thermal conductivity value (which is needed to fulfill divertor requirements). Further, in order to have materials adequate for plasma environment conditions, irradiation with electron beams was performed, and the modification of the exposed surface was evaluated. The thermal expansion behavior was also investigated to verify the possibility of further including the materials in divertor-type components. A composition from the most promising material (W-ZrC) was also produced with larger dimensions and then cut into shapes for 3p-bending tests, which were performed from RT to above 600 °C.

### 2.3. Sample Characterization

The microstructures of the samples were analyzed using a field-emission scanning electron microscope (FESEM) (Zeiss Gemini 500) equipped with a backscattered electron detector. Its sensitivity to Z allows for the identification of different elements even at a nanometric resolution, in contrast to classic EDX analyses. The exposed surfaces of the irradiated samples were investigated using an FEI Inspect S50 SEM with an Everhart–Thornley detector (ETD) to work with secondary electrons (SEs).

The thermophysical properties were investigated using a Laser Flash Analyzer (Netzsch LFA 457 Microflash) from room temperature up to 1000 °C, and the expansion coefficients were determined in the same temperature range using a Netzsch 402 C dilatometer. The LFA equipment allows for the direct measurement of thermal diffusivity, α, by analyzing the temperature variation on one surface of the sample when a calibrated laser pulse is applied to the other surface. The specific heat of the materials, *C*_p_, can be determined by using a differential method using a reference sample (in this case, Mo, NIST certificate SRM781). The thermal conductivity can then be calculated as λ = α × ρ × *C*_p_, where ρ is the density of the sample. The sample density was determined at room temperature by the Archimedes method in water using a high-resolution balance.

Three-point bending tests were carried out with INSTRON 5982 equipment able to apply forces up to 100 kN and equipped with a high-temperature furnace. The tests were performed on samples in the shape of rectangular bars with a size of 20 mm length, 2 mm width and 1.5 mm thickness. A bending load was applied with a speed of 0.05 mm/min.

### 2.4. Sample Irradiation

**Figure 2:**
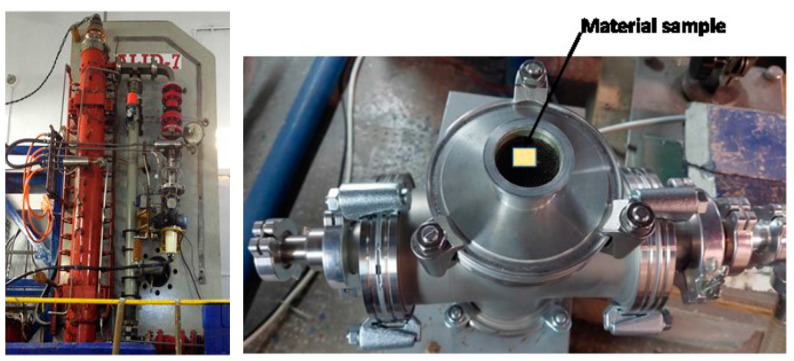
ALID-7 electron beam accelerator (**left**) and the sample chamber (**right**).

Samples of W-D, W-Fe, W-ZrC, W-SiC and W-Cr were irradiated by electron beams of 6 MeV under low-vacuum conditions using the ALID-7 electron beam accelerator at NILPRP (see Figure 2). Each sample was irradiated for 10 min and then left inside the vacuumed chamber for 30 min until cooled to room temperature. No active cooling was applied during irradiation.

The materials were irradiated with a beam fluency of 1 × 10^12^ e^−^/cm^2^ per pulse, and the corresponding charge per electron beam pulse was 8.8 × 10^−7^ C. The peak beam current per pulse was 5.4 × 10^−1^ A, while the current density reached a value of 4.1 × 10^−2^ A/cm^2^. The flux density was 2.5 × 10^17^ e^−^/s/cm^2^. The frequency of the beam was 53 Hz with a pulse duration of 4 μs.

## 3. Results

### 3.1. Density and Microstructure

Usually, for ceramic (hard) dispersions included in a hard metal matrix without a mechanical alloying step and without interactions during the consolidation process, the ceramic material is located between the matrix grains, and taking into account geometric (volume-filling) considerations, at least a part of the dispersion is trapped at the grain boundaries of the matrix grains. In the present case, the samples contain mixtures of particles with very different grain sizes (40 microns and 800 nanometers for W and a few tens of nanometers for the dispersions). Therefore, the main interest is related to answering the following questions: (i) Are enough dispersions located at grain boundaries? (ii) Are the dispersions more concentrated in areas with smaller W grains (that is, following the volume-filling trend) or uniformly distributed? (iii) How does the chemical interaction (as expected for W-SiC and W-diamond) affect the dispersion distribution? To answer these questions, we used a backscattered electron detector (EBS), since this technique is able to provide a Z-sensitive image at a higher resolution in comparison to the classic EDX analysis, which cannot offer a nanometric-scale spatial resolution. Additionally, to prevent possible reactions, the samples were not etched, and to prevent contamination, polishing was performed using diamond grinding plates with a mesh well above the dispersion dimensions.

**Figure 3:**
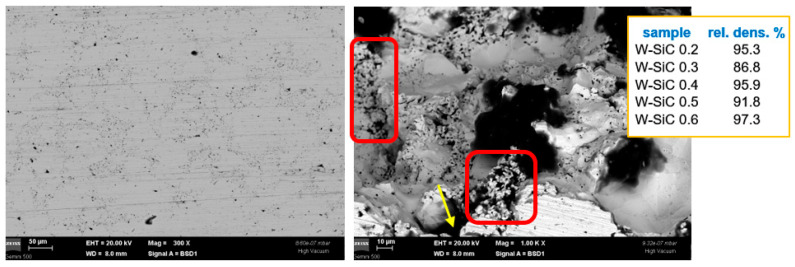
Cross-section SEM images obtained using a backscattered electron detector for W-SiC materials: low magnification (**left**) and higher magnification in a fractured part, also near a larger pore (**right**). Here, the areas marked in red correspond to agglomerations of smaller W grains, while the yellow arrow indicates a pore. The inset summarizes the density values of these materials.

In the case of the W-SiC material, as illustrated in Figure 3, in spite of the apparent uniform distribution of the dispersions at the W grain boundaries (Figure 3, left), some areas seem to have a larger content of dark dots, which might be connected to agglomerations of smaller W grains. Since the EBS is also sensitive to the depth of the surface, a more detailed analysis is required to discriminate between SiC grains and pores. To clarify the situation, an image with a larger magnification obtained in a fractured part of the sample (Figure 3, right) shows that SiC is more agglomerated in areas with smaller W grain sizes (the areas marked in red). Here, larger pores can also be observed (e.g., the one marked by the yellow arrow). Moreover, the density of the materials has no clear correlation with the dispersion content, possibly due to the W-SiC interaction and the formation of W-C and W-Si compounds. This reaction has already been documented for SiC matrix-W-dispersion composites [44] but, in the present case, leads to the local contraction of W-SiC-reacted material and the generation of pores.

**Figure 4:**
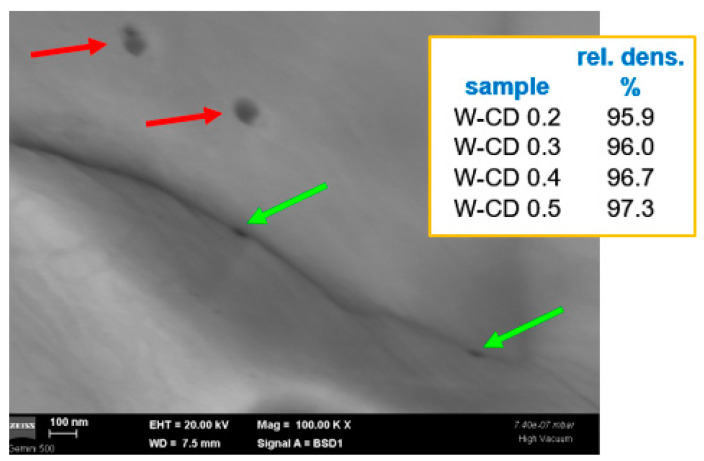
Cross-section SEM image obtained using a backscattered electron detector for W-diamond materials: the green arrows indicate the dispersion located at grain boundaries, while the red ones indicate the dispersion located on the grains. The inset summarizes the density values of these materials.

The diamond dispersions seem to be distributed both at W grain boundaries and on the grains (see Figure 4). It also appears, as expected, that C has reacted with W, forming W-carbides. In this image, the diamond inclusions appear different in terms of dimensions and colors, possibly suggesting different formations. However, since diamond grains are very small (APS 10 nm), they are more difficult to find, especially at high magnifications. Thus, the explanation for the morphological differences from the selected image is most likely due to random factors (distribution and size of diamond grains’ agglomerations). These dispersions are also too small to act as grain growth inhibitors, and larger diamond grains, even if they do not completely dissolve in W, will be graphitized under neutron irradiation. However, the samples have good densities, which increase with the diamond content. For W samples with larger WC insertions, improved mechanical properties have been observed [45].

**Figure 5:**
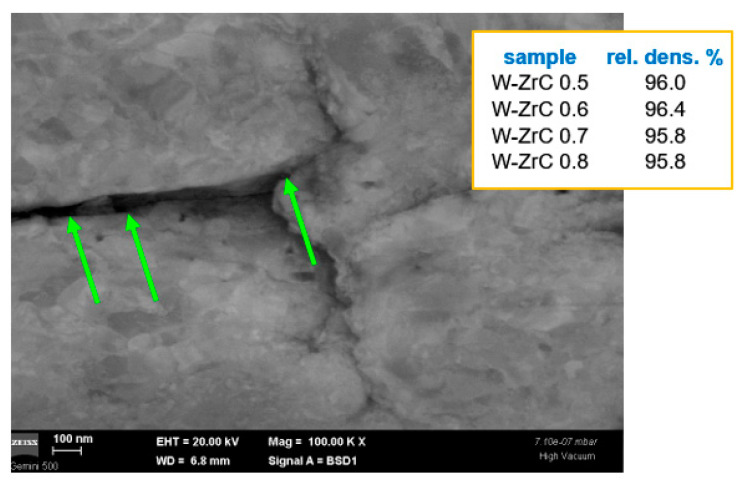
Cross-section SEM image obtained using a backscattered electron detector for W-ZrC materials. The inset summarizes the density values of these materials.

In the case of W materials with ZrC dispersions, as shown in Figure 5, one can observe the dispersion distributed at the W grain boundaries (marked by green arrows). Sharp intergrain surfaces suggest a possible effect of the dispersion even in the SPS consolidation process. The samples also have good densities; however, lower ZrC concentrations seem to provide higher density values. All of these results could in fact be expected, taking into account the work of Z.M. Xie et al. [40].

**Figure 6:**
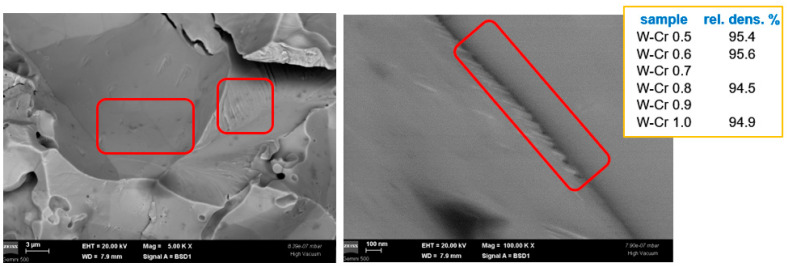
Cross-section SEM images obtained using a backscattered electron detector for W-Cr materials. Darker small spots on the W grain surface, as identified in the red frames (**left**), result from W-Cr solid solution formation, and similar smaller points are also detected between grains as seen in the red frame (**right**). The inset summarizes the density values of these materials.

In the case of W-Cr materials, the nanometric Cr dispersions distributed at the W grain boundaries and also Cr agglomerations, as identified in the red frames, appear to have reacted with W, as expected (see Figure 6), and may have produced a similar blocking effect. To clarify this, HRTEM investigations are needed. However, their present sizes might also be too small for the expected effect; thus, in future work, larger dimensions should be considered. The densities at higher dispersion contents are also decreased, suggesting that further material optimization is needed.

**Figure 7:**
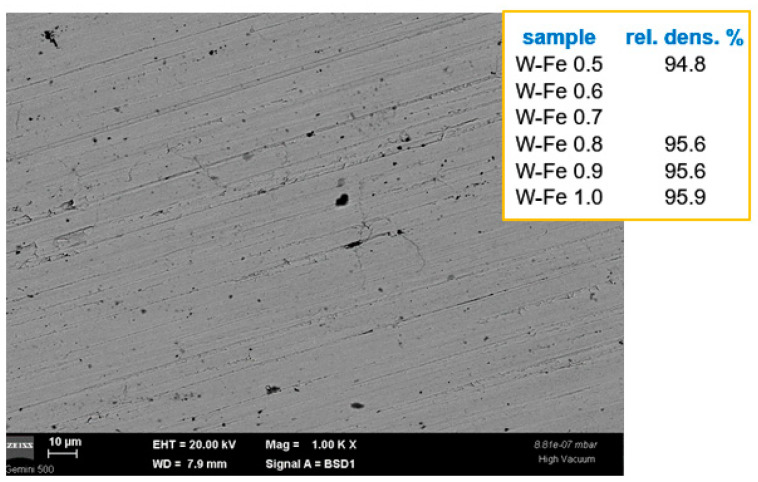
Cross-section SEM image obtained using a backscattered electron detector for W-Fe materials. Dispersion location at W grain boundaries can be observed, together with the presence of pores. The inset summarizes the density values of these materials.

The Fe dispersions (see Figure 7) are distributed between W grains and at W grain boundaries, as expected from the melting of Fe during the SPS consolidation. In contrast to the case of W-SiC materials, here, the W grains can only barely be seen. This can be explained by the smaller volume of Fe and also by its melting. As some preliminary investigations have shown, for samples with about a 4% volume of micrometric Fe dispersions in a micrometric grain W matrix, pools of Fe should be easily observed. In the present case, on the one hand, the lower Fe content could be mostly hidden in areas with nanometric W grains, and on the other hand, as also observed in the previous experiments, due to the reduction in the Fe-specific volume upon cooling, we can presume that Fe remains glued to W and generates pores. Indeed, some large pores can be also observed, and the emergence of Fe at the exposed surface during irradiation experiments (see Section 3.3) might be proof of this assumption. However, the concentrations seem to be too small to also allow for an expected “coating” of W grains, therefore avoiding possible pseudo-ductility generated by that behavior. Additionally, only the higher concentrations provide acceptable densities.

### 3.2. Thermophysical Properties

**Figure 8:**
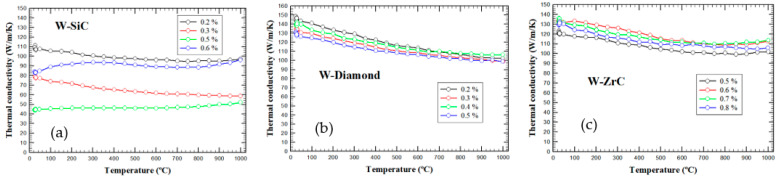
Temperature-dependent thermal conductivity of W-ceramic composites for different dispersion concentrations: (**a**) W-SiC materials, (**b**) W-diamond materials and (**c**) W-ZrC materials.

A value of at least 100 W/m/K for the thermal conductivity of a divertor armor material is required in the DEMO reactor design, assuming a steady-state operation, with the exposed surface reaching values close to about 1000 °C. For the SPS-ed W materials with ceramic dispersions, the temperature-dependent thermal conductivities are summarized for different dispersion contents in Figure 8.

For W-SiC materials, low-density specimens also have low thermal conductivity values (Figure 8a), but no clear trend could be determined between sample densities and thermal conductivity values or between specific heat (see Figure S1 in the Supplementary File) and thermal conductivity. However, the thermal conductivity data follow the trend of the thermal diffusivity (see Figure S2 in the Supplementary File), which is dependent on the sample morphology at both microscopic and macroscopic scales. Taking into account that thermal conductivity is calculated as λ = α × ρ × C_p_, an almost constant thermal diffusivity combined with an increase in specific heat for increasing temperatures leads to an increase in thermal conductivity values upon a temperature increase. A physical explanation might be assumed from the following grounds: (i) the thermal conductivity of a usual material is given by the sum of the electronic and lattice contributions; (ii) at high temperatures, the electrons’ contribution to thermal conductivity is almost constant with the temperature (Wiedeman Franz law), while the lattice contribution is determined mostly by phonon Umklapp processes, i.e., a 1/T temperature dependence, leading to an overall 1/T behavior; and (iii) in the case of many point-like defects, J. Callaway and H.C. Von Baeyer [46] proposed a negative linear dependence on 1/T for the lattice contribution, which was able to explain the increase in the thermal conductivity with increasing temperatures for various disordered oxides. Similar thermal conductivity behavior was observed also for some W-Cu materials [20] and Cu-ceramic composites [22,23]. In the W-SiC materials, we also expect [44] a W + SiC reaction that forms W-Si and W-C compounds, which might also depend on the W grain sizes and their distribution in the sample. The thermal transport phenomenology for such composite materials is rather complex (see, e.g., [47]) and exceeds the scope of the present work. Nevertheless, all specimens are unacceptable from the point of view of the 100 W/m/K limit imposed for a divertor material. In contrast, in the case of W-diamond (Figure 8b) and W-ZrC (Figure 8c) materials, all concentrations tested show thermal conductivity values above the required limit. This is a promising behavior, taking into account that the materials are not optimized from the point of view of density. Moreover, for both types of materials, the thermal conductivity, thermal diffusivity and specific heat values show small variations at different concentrations (see, e.g., Figures S3 and S4 in the Supplementary File for W-ZrC specific heat and thermal diffusivity, respectively).

**Figure 9:**
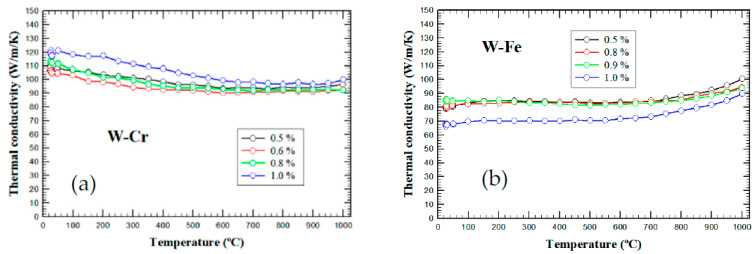
Temperature-dependent thermal conductivity of W-metal composites for different dispersion concentrations: (**a**) W-Cr materials and (**b**) W-Fe materials.

The temperature-dependent thermal conductivities are summarized in Figure 9 for the two types of W-metal composites. Again, using the 100 W/m/K rule, it can be concluded that for W-Cr materials, the thermal conductivity values are acceptable, while for W-Fe materials, the obtained values are too low for divertor applications. It is interesting to note that the lowest thermal conductivity value for W-Fe materials is obtained for the sample with the highest concentration of Fe, which has also the highest density. This might indicate that the W-Fe interfaces are responsible for decreasing the thermal conductivity, as can also be deduced for the thermal diffusivity values, which, in this case, are less than 80% of the value measured for the lowest Fe concentration. One can also observe that, in the case of W-Fe materials, the thermal conductivity temperature dependence deviates from the classic 1/T behavior expected for metallic materials. Although slight Fe-W interdiffusion cannot be completely excluded, we assume that the almost-flat behavior is determined by the W-Fe interface effects. Indeed, the thermal boundary conductance [48] is expected to be almost constant at temperatures above 300–400 K, and there is also a dependence on the interfacial bonding strength. This can explain the additional increase in the thermal conductivity at temperatures approaching 1000 °C, where the thermal expansion of Fe increases the pressure on adjacent W grains. On the other hand, for the W-Cr materials, the highest values for thermal conductivity are obtained with the highest Cr concentration, suggesting, as also observed from the microstructure investigations, that either the Cr content or the Cr particle dimensions used in this study are not sufficient to produce a better material.

**Figure 10:**
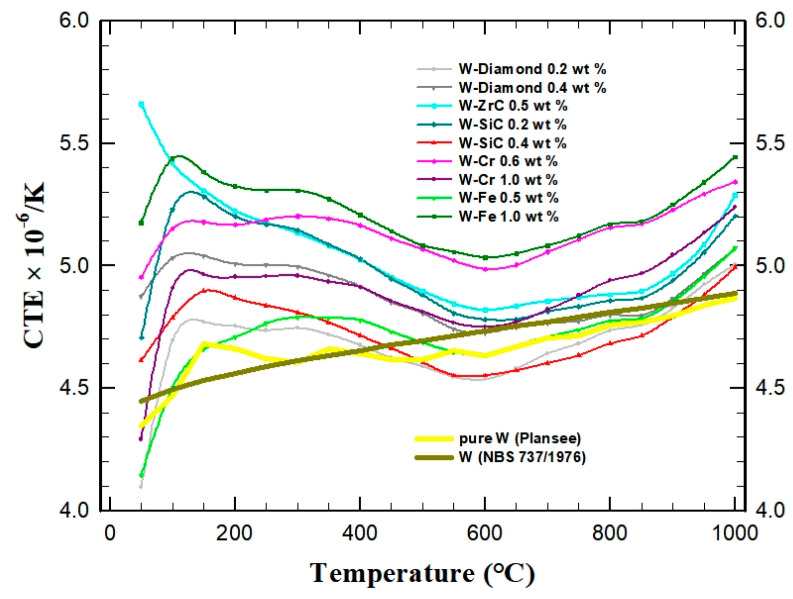
Temperature-dependent CTEs of selected W−dispersion composites. For comparison, values obtained for typical pure W materials are also included.

The dilatometry measurements performed on some of the samples show, as illustrated in Figure 10, that the thermal expansion of all materials is not significantly affected by the dispersions, with the values being very close to that of pure W. In fact, a survey of the derived CTEs shows that, for most of the composites, the values are slightly higher than that for pure W, which can help to reduce thermal-induced stresses in components with Cu and/or steel parts.

### 3.3. Post-Irradiation Analyses of Exposed Surfaces

**Figure 11:**
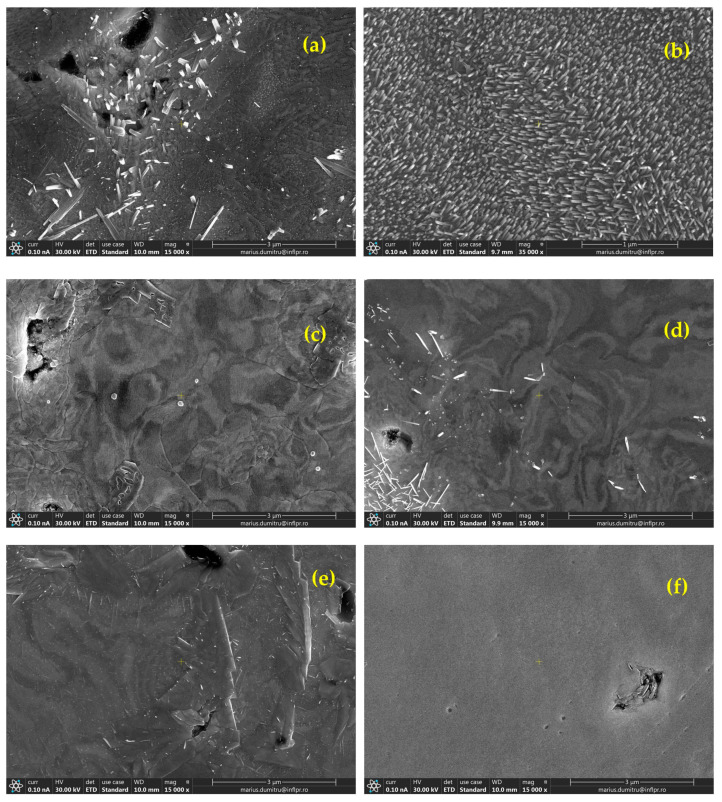
SEM images, obtained using a secondary electron detector, of the exposed surfaces of different W-ceramic materials after 6 MeV electron irradiation: (**a**) W-SiC 0.2%, (**b**) W-SiC 0.4%, (**c**) W-diamond 0.2%, (**d**) W-diamond 0.4%, (**e**) W-ZrC 0.5% and (**f**) W-ZrC 0.6.

**Figure 12:**
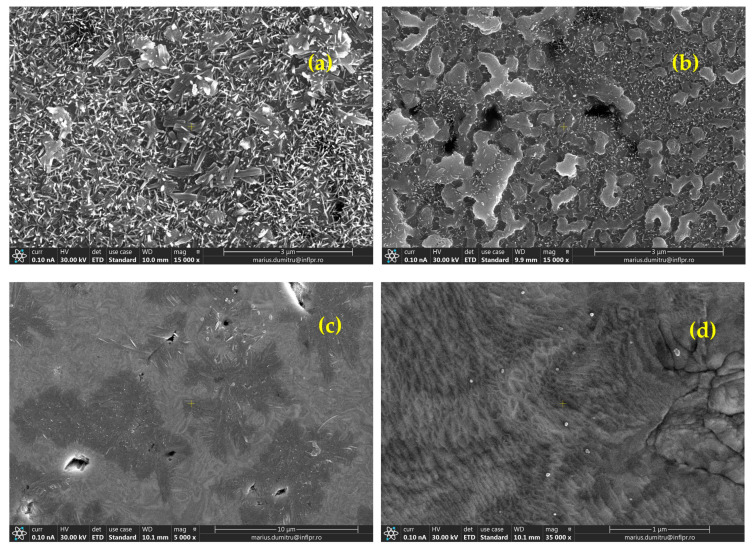
SEM images, obtained using a secondary electron detector, of the exposed surfaces of different W-metallic materials after 6 MeV electron irradiation: (**a**) W-Cr 0.6%, (**b**) W-Cr 1.0%, (**c**) W-Fe 0.5% and (**d**) W-Fe 1.0%.

Figure 11 and Figure 12 summarize the sample surface morphologies after irradiation with 6 MeV electrons (flux density was 2.5 × 10^17^ e^−^/s/cm^2^) for W-ceramic and W-metallic composites, respectively. With these values and in the absence of active cooling of the surface, taking into account the theoretical loss of energy for the electrons in pure W (thus neglecting the dispersions’ presence) of 16.8 MeV/mm, we can assume that all of the energy is absorbed in about 350 microns from the exposed surface, and this leads to a possible surface temperature of up to around 1800 °C during irradiation. Of course, this is a crude estimation, and the materials should have different behaviors depending on their properties and also on the electron beam inhomogeneity at the microscopic scale.

For W-SiC (Figure 11a,b) and W-diamond materials (Figure 11c,d), strong reactions at the heated surface (exposed to e^−^ flux) and crystal formation (W + C + Si and W + C, respectively) can be observed in the SEM images. The number of crystalline formations on the exposed surface increases with the dispersion concentrations. On the other hand, for W-ZrC materials (Figure 11e,f), an overall good behavior was detected, with only small defects on the exposed surface and clearly without large-crystal formation. For W-Cr materials (Figure 12a,b), strong reactions at the heated surface (exposed to e flux), repeated melting and crystal formation (W + Cr + O) can be observed, reminiscent of the behavior observed for self-passivating W alloys. However, the low concentration of Cr and the absence of Y will probably not be the best premises for effective protection (note that also the amount of O in the irradiation chamber was reduced). For W-Fe materials (Figure 12c,d), the images of the exposed surfaces show evidence of the repeated melting of Fe, which was expelled on the surface (almost the entire surface is covered with Fe).

## 4. Discussion

Based on microstructural and thermophysical and their evolution under e- irradiation, ZrC- and Cr-containing materials have been selected for further investigations, while the other three dispersions have been deprioritized. Taking into account this selection, new samples have been produced for mechanical tests.

**Figure 13:**
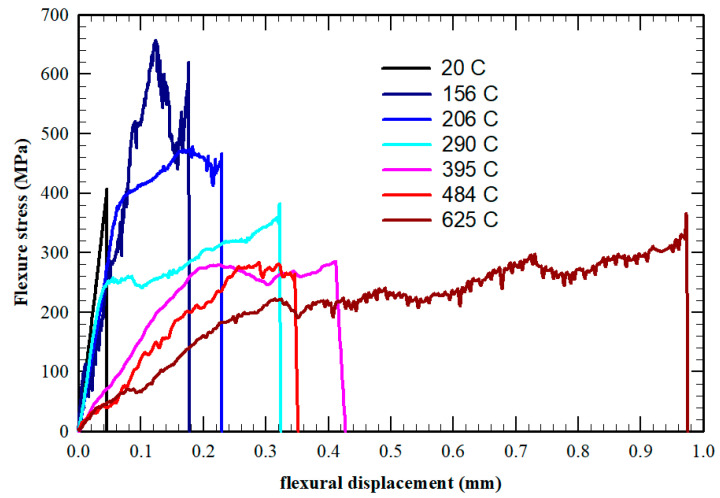
Three-point bending behavior at different temperatures for W-ZrC (0.5 wt.%) composites.

The first preliminary results obtained on W-ZrC 0.5 wt.% are plotted in Figure 13 for different temperatures: 20, 156, 206, 290, 395, 484 and 625 °C. Surprisingly, even the sample tested at 156 °C shows some ductility in these tests, as do all of the other samples tested at higher temperatures, with curves and values similar to those obtained by Xie et al. [41] on similar SPS-ed samples but, in this work, at much lower temperatures, approaching the results obtained by the same group [40] on hot-rolled W-ZrC.

Even more surprisingly, most of the samples maintained macroscopic integrity in spite of severe cracking during the test.

Post-mortem SEM analyses are ongoing and should clarify the type of fractures produced. The tests will also be repeated with other similar materials in order to verify the reproducibility and understand the role of the dispersions. Moreover, the materials’ quality can be significantly improved by improving the SPS process and subsequent thermo-mechanical treatment.

## 5. Conclusions

A screening study was performed to investigate the effects produced by metallic and ceramic nanometric dispersions in W. Based on microstructural and thermophysical properties and their evolution under e^−^ irradiation, ZrC- and Cr-containing materials have been selected for further investigations, while SiC-, diamond- and Fe-containing materials have been deprioritized. For W-ZrC (0.5 wt.%), promising mechanical properties have already been observed in temperature-dependent 3p bending tests.

## Figures and Tables

**Table 1:** Summary of the test materials’ compositions.

Material	No. of Samples	wt.% Concentration	vol.% Concentration
W-SiC	5	0.2–0.6	1.19–3.49
W-ZrC	4	0.5–0.8	1.42–2.27
W-D	4	0.2–0.5	1.08–2.68
W-Cr	6	0.5–1.0	1.34–2.65
W-Fe	6	0.5–1.0	1.21–2.41

## Data Availability

The data presented in this study are available on request from the corresponding author.

## Supplementary Materials

The following supporting information can be downloaded at https://www.mdpi.com/article/10.3390/nano13061012/s1. Figure S1: Specific heat of the W-SiC composites; Figure S2: Thermal diffusivity for W-SiC composites; Figure S3: Specific heat of the W-ZrC composites.; Figure S4: Thermal diffusivity for W-ZrC composites.
