# Supplementary material for: Microengineering Design for Advanced W-Based Bulk Materials with Improved Properties
Source: Nanomaterials (Basel). 2023 Mar 11;13(6):1012. doi: 10.3390/nano13061012 (PMC10056536; doi:10.3390/nano13061012)
Supplement: Supplementary file 1 [file nanomaterials-13-01012-s001.zip › nanomaterials-2223611-supplementary.pdf]

nanomaterials

Supplementary Materials

Microengineering Design for Advanced W-Based Bulk Materials with Improved Properties

Magdalena Galatanu [1], Monica Enculescu [1], Andrei Galatanu [1,*], Dorina Ticos [2], Marius Dumitru [2] and Catalin Ticos [2]

[1] National Institute of Materials Physics, Atomistilor Street 405 A, Magurele, 077125 Ilfov, Romania
[2] National Institute for Laser, Plasma and Radiation Physics, Atomistilor Street 409, Magurele
* Correspondence: gala@infim.ro

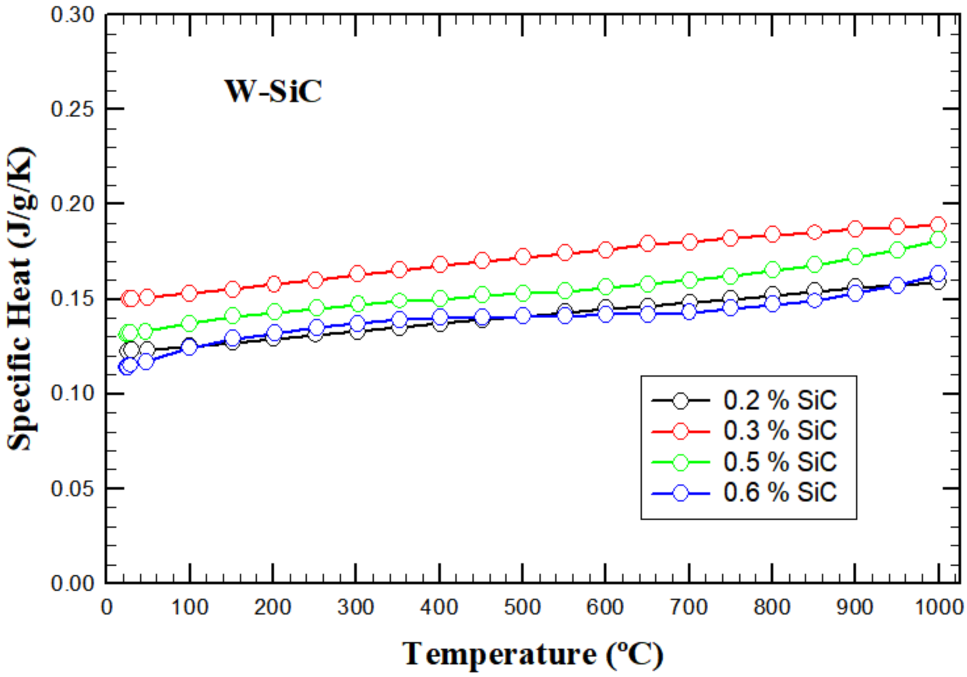

Figure S1. Specific heat of the W-SiC composites.

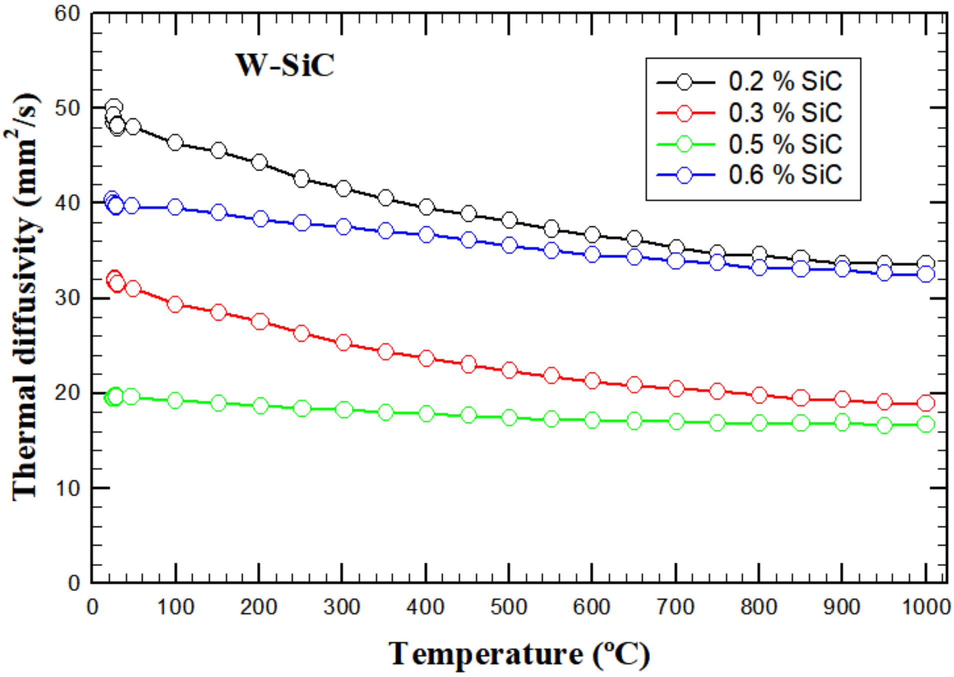

Figure S2. Thermal diffusivity for W-SiC composites.

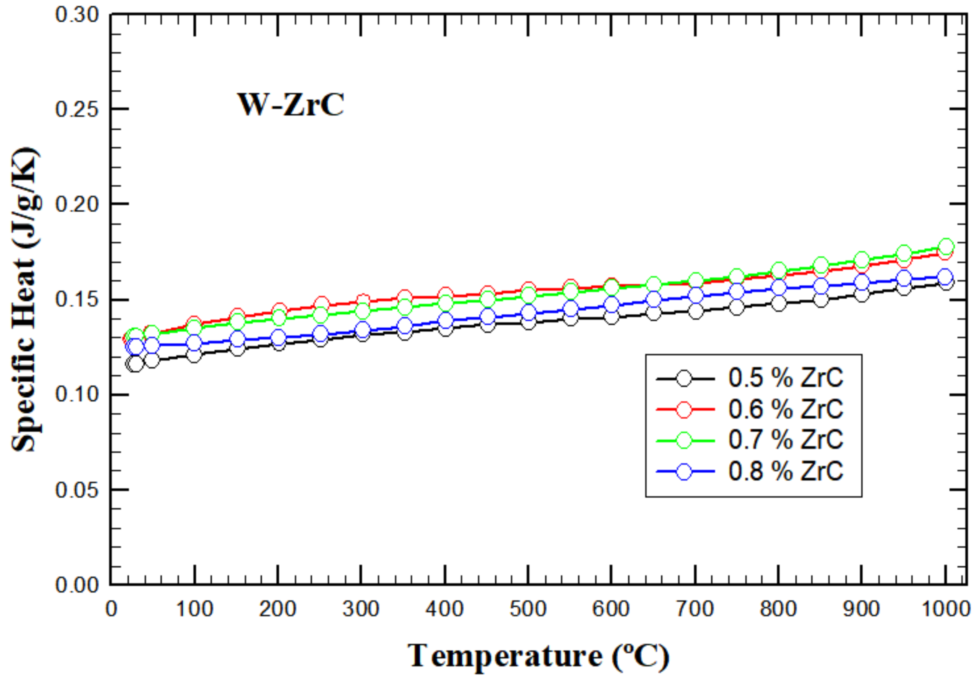

Figure S3. Specific heat of the W-ZrC composites.

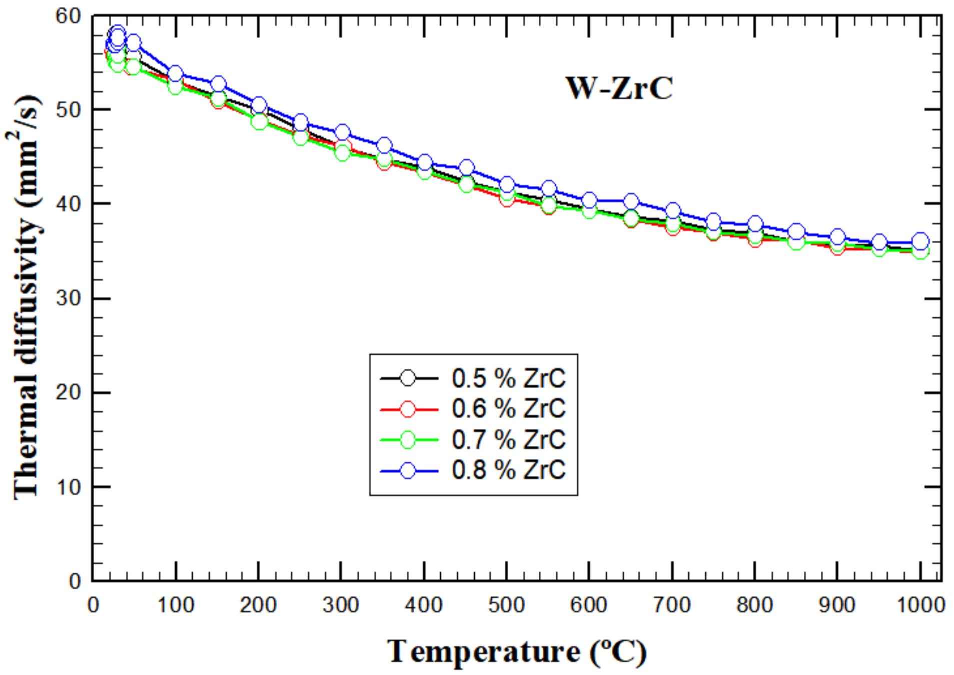

Figure S4. Thermal diffusivity for W-ZrC composites.
